# USP14 as a novel prognostic marker promotes cisplatin resistance via Akt/ERK signaling pathways in gastric cancer

**DOI:** 10.1002/cam4.1770

**Published:** 2018-09-17

**Authors:** Ying Fu, Gang Ma, Guolong Liu, Bin Li, Hui Li, Xishan Hao, Liren Liu

**Affiliations:** ^1^ Department of Gastrointestinal Cancer Biology Tianjin Medical University Cancer Institute & Hospital National Clinical Research Center for Cancer; Key Laboratory of Cancer Prevention and Therapy Tianjin; Tianjin’s Clinical Research Center for Cancer Tianjin 300060 China; ^2^ Department of Gastrointestinal Surgery Tianjin Medical University Cancer Institute & Hospital National Clinical Research Center for Cancer; Key Laboratory of Cancer Prevention and Therapy Tianjin; Tianjin’s Clinical Research Center for Cancer Tianjin 300060 China

**Keywords:** chemoresistance, deubiquitylases, gastric cancer, USP14

## Abstract

Gastric cancer (GC) ranks the third leading cause of global cancer mortality. Despite recent progress in surgery combined with chemotherapy, the outcomes of GC patients have barely improved. Therefore, better understanding of the molecular mechanisms involved in chemoresistance of GC may help develop novel strategies to treat this deadly disease. Previous evidence has shown aberrant expressions of USP14 in multiple malignancies, suggesting an important role of USP14 in tumorigenesis. However, its role in modulating chemoresistance in GC still remains elusive. In this study, we observed that USP14 levels were significantly increased in GC tissues compared to the paired normal tissues. Multivariate analysis demonstrated that USP14 level was an independent prognostic factor for DFS in GC patients. Silencing of USP14 promoted proteasomal degradation of p‐ERK (T202/Y204) and p‐Akt (T308/S473), thus inactivating Akt and ERK signaling pathways. Interestingly, silencing of USP14 alone was not sufficient to cause overt effects on cell growth, proliferation, and apoptosis, while resulting in significant apoptosis in the presence of cisplatin in GC cells. Thus, knockdown of USP14 sensitized GC cells to cisplatin by triggering cisplatin‐induced apoptosis via impeding Akt and ERK signaling pathways. These results revealed a novel role of USP14 in modulating chemosensitivity of GC cells, suggesting USP14 may serve as not only a prognostic marker, but also a potential therapeutic target for GC patients.

## 1 BACKGROUND

Gastric cancer (GC) is the third most common cancer worldwide.^1^ Most GC patients are diagnosed in East Asia. In China alone, 679 000 new cases and 201 000 deaths are reported in 2015.^2^ Although new therapeutically strategies consisting of surgery, chemoradiation, and biotherapy have been developed, the overall survival of GC patients, especially those in advanced stages, still remains low. Thus, to reveal the molecular mechanisms underlying tumor progression against treatment may help to develop novel therapeutics, thereby improving outcomes of GC patients.

Protein homeostasis is essential in regulating diverse cellular functions. Two major systems, proteasomes and lysosomes systems, dictate the degradation of unnecessary proteins in cells.^3^ The proteasome system contains E1 activating enzymes, E2 conjugating enzymes, and E3 ligases, which may cooperate to link ubiquitin chain to the Lys48 residue of the targeting proteins for degradation.^3^ On the other hand, the primary function of the deubiquitylases (DUBs) is to rescue proteins from degradation by removing the “death tags” from them, and meanwhile maintaining the cellular mono‐ubiquitin pool.^4^ Mammalian DUBs consist of around 90 members, which can be divided into ubiquitin‐specific protease (USP), ubiquitin C‐terminal hydrolase (UCH), ovarian tumor (OTU), Machado‐Joseph disease (MJD), and Jab1/Mpn/Mov34 (JAMM) subfamily.^4^ As DUBs are important in controlling protein turnover, it is not surprised to find dysfunction of DUBs in cancer. Recent studies show that DUBs play essential roles in proliferation, motility, and chemoresistance of malignant cells through stabilizing critical proteins.^5^ For example, USP49 deubiquitinates FKBP51, which serves as a scaffold for dephosphorylating Ser473 residue at Akt, and further sensitizes pancreatic cancer cells to gemcitabine.^6^ In spite of the advances in recent years, the functions of DUBs in cancer biology remain largely unknown.

USP14, a member of USP deubiquitylases subfamily, is functionally associated with 26S proteasome.^4^ USP14, highly expressed in brains, is critical for normal cerebral functions, as abnormal brain morphology and dysfunctional synaptic transmission have been observed in Usp14^ax‐J/ax‐J^ mice.^7^ Increasing evidence has shown that USP14 is also implicated in the progression of various cancers.^5^ Depleting USP14 using shRNAs in ovarian cancer cells or blocking USP14 activity with the inhibitor b‐AP1 in breast cancer cells can inhibit proliferation and promote apoptosis in those cells.^8^, ^9^ Recently, USP14 is reported to interact with and thus stabilize vimentin protein, thereby promoting the proliferation and motility of GC cells.^10^ However, the role of USP14 in modulating chemosensitivity in GC remains elusive.

In this study, we observe higher expression levels of USP14 in GC samples compared to the paired normal tissues and that increased USP14 level is associated with poor prognosis and higher recurrence rates in GC patients. Furthermore, our results show that knockdown of USP14 expression sensitizes GC cells to cisplatin through impeding Akt or ERK signaling pathway, while having little impact on the proliferation of these cells. Thus, we reveal a novel role of USP14 in modulating chemosensitivity of GC cells, implying USP14 may serve as a prognosis indicator and a potential therapeutic target for GC patients.

## 2 METHODS

### 2.1 Patients and GC tissue samples

Fresh GC tissues and adjacent normal tissues, which were located at least 5 cm away from the primary lesions, were collected from 23 GC patients who received radical resection at the Department of Gastrointestinal Surgery in Tianjin Medical University Cancer Institute and Hospital (Tianjin, China). Upon resection, these samples were stored in a liquid nitrogen container till extracting RNAs and proteins. A total of 113 GC patients receiving radical surgery and following cisplatin‐based chemotherapy throughout 2012 were enrolled in this study. All these patients did not receive neo‐adjuvant chemoradiation therapy, and the relevant clinical data were available. The resected GC samples from these patients were confirmed histologically. A GC tissue chip containing 15 paired of malignant and adjacent normal tissues was obtained from Shanghai Outdo Biotech (Shanghai, China). All experiments involving GC tissue samples were approved by the ethics committee of Tianjin Medical University Cancer Institute and Hospital.

### 2.2 Cell lines and reagents

Gastric cancer cell line KATO III was obtained from ATCC (Manassas, VA, USA) and cultured in IMDM with 20% FBS. GC cell line MKN45 was obtained from National Infrastructure of Cell Line Resource (PUMC, Beijing, China) and cultured in RPMI1640 with 10% FBS. All cells were maintained under humanized condition (37°C, 5% CO_2_) and continual culture did not exceed 2 months. Cisplatin (5 mg/mL) was purchased from Hansoh Pharmaceutical Group Co., Ltd. (Jiangsu, China). MG132, PD98059, and LY294002 were purchased from Cell Signaling Technology (Danvers, MA, USA). Nontarget and USP14‐targeting siRNAs were purchased from RiboBio Co., Ltd. (Guangdong, China). The target sequences of siRNAs against USP14 are as follows: 5‐CTGGCATATCGCTTACGTT‐3, 5′‐TCCAGTATTCCACCTATTA‐3′, 5′‐TTGCCGAGAAAGGTGAACA‐3′.

### 2.3 Antibodies

Rabbit anti‐USP14 mAb (#11931), anti‐pan‐Akt mAb (#4691), anti‐p‐Akt (T308) mAb (#4056), anti‐p‐Akt (S473) mAb (#4060), anti‐pan‐ERK mAb (#4695), anti‐p‐ERK (T202/Y204) mAb (#4376), anti‐LC3A/B mAb (#12741) were purchased from Cell Signaling Technology. Mouse anti‐GAPDH mAb (G8795) was purchased from Sigma (Shanghai, China). Rabbit anti‐PARP mAb (ab32561) and mouse anti‐PCNA mAb (ab29) were purchased from Abcam (Cambridge, UK). Rabbit anti‐USP14 mAb (#11931) were obtained from Proteintech (Wuhan, Hubei, China).

### 2.4 In silico analysis of USP14 in GC datasets

GEO dataset GSE27342 was used to screen 46 DUBs’ expressions. USP14 expressions were further analyzed in three additional GC datasets (GSE29272, GSE33335, and GSE63089). The correlations between USP14 expression and overall survival of GC patients were assessed using Kaplan‐Meier Plotter based on TCGA data.^11^

### 2.5 Transfection and treatment of drugs

For transient knockdown of USP14 expression, KATO III or MKN45 cells were seeded in 6‐well plates and transfected with nontarget or USP14‐targeting siRNA pool using HiPerFect (Qiagen, German) according to the manufacturer's instruction. The working concentration of siRNAs was 100 nmol/L. MG132 (10 μmol/L) was incubated with KATO III or MKN45 cells for 4 hours to inhibit the function of proteasome‐degradation system. Before incubated with cisplatin for 24 hours, KATO III or MKN45 cells were pretreated with LY294002 (10 μmol/L) and/orPD98059 (10 μmol/L) for 1 hour to block the transduction of Akt and ERK signaling pathway, respectively.

### 2.6 WST‐1 assay

MKN45 (5 × 10^3^/well) and KATO III (8 × 10^3^/well) cells were seeded in 96‐well plates, respectively. The viability of these cells was tested using WST‐1 (Roche, Basel, Switzerland) following the manufacturer's instruction. In every single assay, sextuple wells for each concentration point of cisplatin were tested and IC50s were calculated accordingly. Two independent assays were performed for MKN45 or KATO III cells, respectively, and representative IC50 and cell viability for each cell line were shown.

### 2.7 RNA and RT‐qPCR

RNA was extracted using TRIzol (Thermo Fisher Scientific, Waltham, MA, USA), and cDNAs were synthesized using PrimeScript™ RT reagent Kit (TaKaRa, Beijing, China). The qPCR assays were performed using SYBR® Premix Ex Taq™ II (TaKaRa) on QuantStudio™ 5 Real‐Time PCR System (Thermo Fisher Scientific). GAPDH and B2M were used as the internal control simultaneously. One representative result of two independent assays was shown.

### 2.8 Western blotting

Cell lysis buffer (100 mmol/L NaCl, 10 mmol/L EDTA (pH 8.0), 50 mmol/L Tris‐Cl (pH 8.0) and 0.5% (v/v) Triton X‐100) with EDTA‐free complete protease and phosphatase inhibitors (Roche) was used to extract proteins. Lysates were separated on 10% SDS‐PAGE gel and transferred to PVDF membranes. The targets were detected using Amersham Imager 600 (GE, Boston, MA, USA). GAPDH was used as the loading control.

### 2.9 Apoptosis and proliferation analysis using flow cytometry

Apoptosis and *proliferation* of KATO III or MKN45 cells were analyzed using Annexin V‐FITC Apoptosis detection Kit II (BD) and BrdU Cell Proliferation Assay Kit (BD, Franklin Lakes, NJ, USA), respectively. The results were measured on FACS Canto II (BD). Representative results of independent assays were shown.

### 2.10 Immunohistochemistry

The immunohistochemistry analyses were performed as described previously.^12^ The results were assessed in an intensity and proportion score‐dependent manner.^12^ In general, the levels of USP14 were calculated by multiplying its staining intensities by the areas of positive staining. The intensities were scored as 0 for negative, 1 for light yellow, 2 for yellowish brown, and 3 for brown. The positive areas were scored as 0 for 0%‐5% positive, 1 for 5%‐25% positive, 2 for 26%‐50% positive, 3 for 51%‐75% positive, and 4 for 75%‐100% positive. According to the calculation results, GC samples were categorized into the low expression group (scored as 0‐3 points) and the high expression group (scored as 4‐12 points). Two pathologists scored the USP14 levels in GC samples independently.

### 2.11 Statistical analysis

The significance of USP14 expression in four GC datasets was analyzed using paired *t* test. The Kaplan‐Meier survival curve was used to assess the correlation between USP14 expression and Disease‐free survival (DFS) of GC patients, and the log‐rank test was used to determine statistical significance. A Cox proportional hazard model was used to examine the risk ratio with simultaneous contributions from several covariates. Other data were analyzed using Student's *t* test. All statistical analyses and graphs drafting were performed via GraphPad Prism 6 (La Jolla, CA, USA). *P*‐value <0.05 was considered statistically significant.

## 3 RESULTS

### 3.1 In silico screen identified USP14 as a candidate prognostic marker in GC

**Figure 1:**
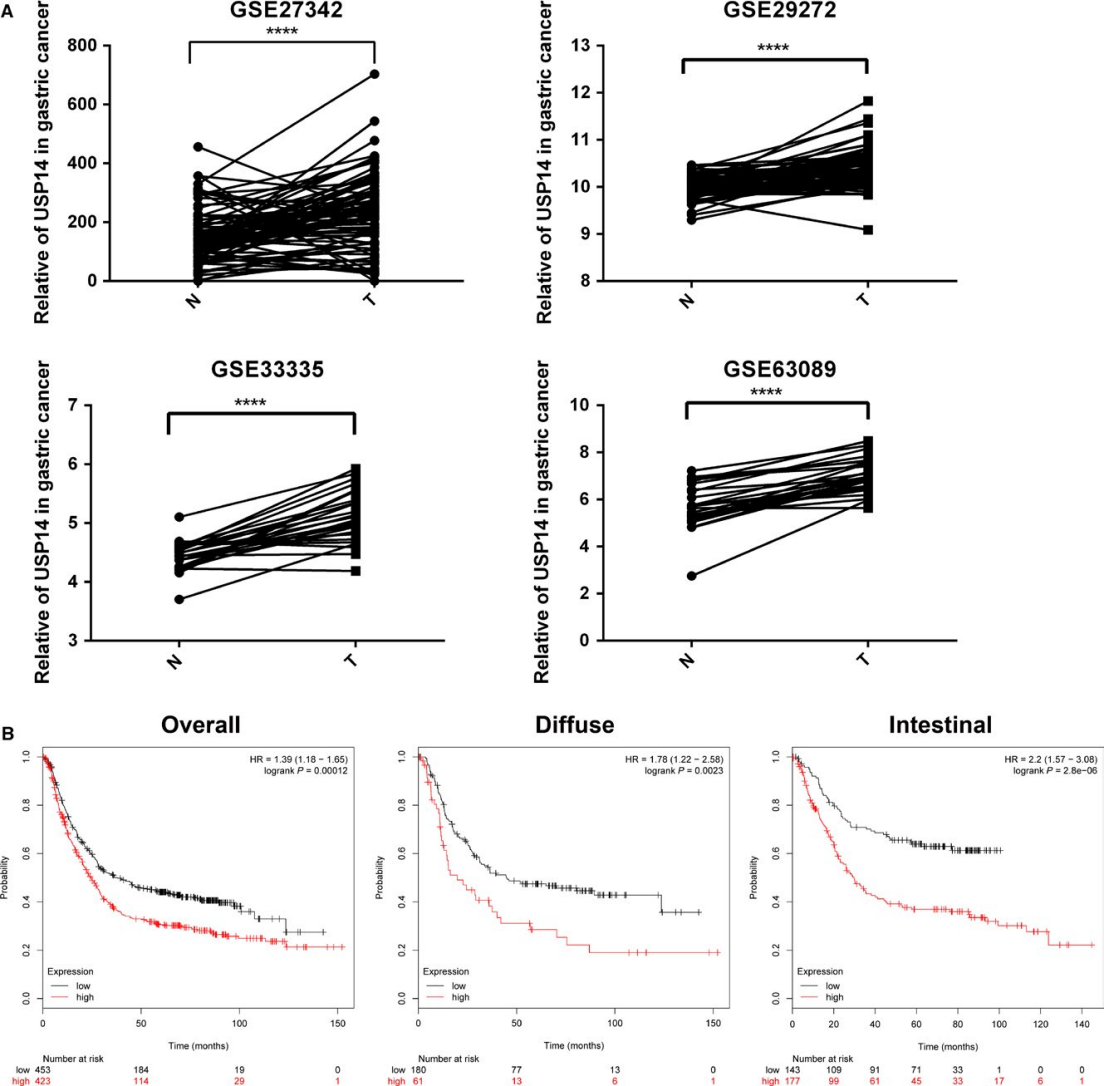
Clinical characteristics of USP14 expression in GC in database. A, Mining four GC datasets (GSE27342, GSE33335, GSE29272, and GSE63089) showed that USP14 expression was higher in GC samples compared with the adjacent normal tissues. Data, ***P <* *0.005, ****P <* *0.0001. B, Increased USP14 expression was associated with the poor OS in diffuse (*P* = 0.0023) or intestinal (*P* = 2.8e‐6) GC patients. This analysis was performed using Kaplan‐Meier Plotter

We first performed an expression screening for all 46 DUBs in a dataset deposited in GEO (GSE27342), among which we found USP14 mRNA levels were significantly increased in the GC samples compared to the adjacent normal tissues (Figures 1A and S1). Then, we assessed USP14 expressions in three additional datasets (GSE29272, GSE33335, and GSE63089) (Figure 1A). Notably, as shown in Figure 1B, aberrantly increased USP14 expression positively correlated with poor overall survival (OS) of GC patients (logrank *P* = 0.00012), and in different pathological subtypes (diffuse, logrank *P* = 0.0023; intestinal, logrank *P* = 2.8e‐6).^11^ Together, these in silico study results showed that USP14 may serve as an OS marker for GC patients, implying important roles it may play in GC.

### 3.2 USP14 expression levels were increased in GC samples

**Figure 2:**
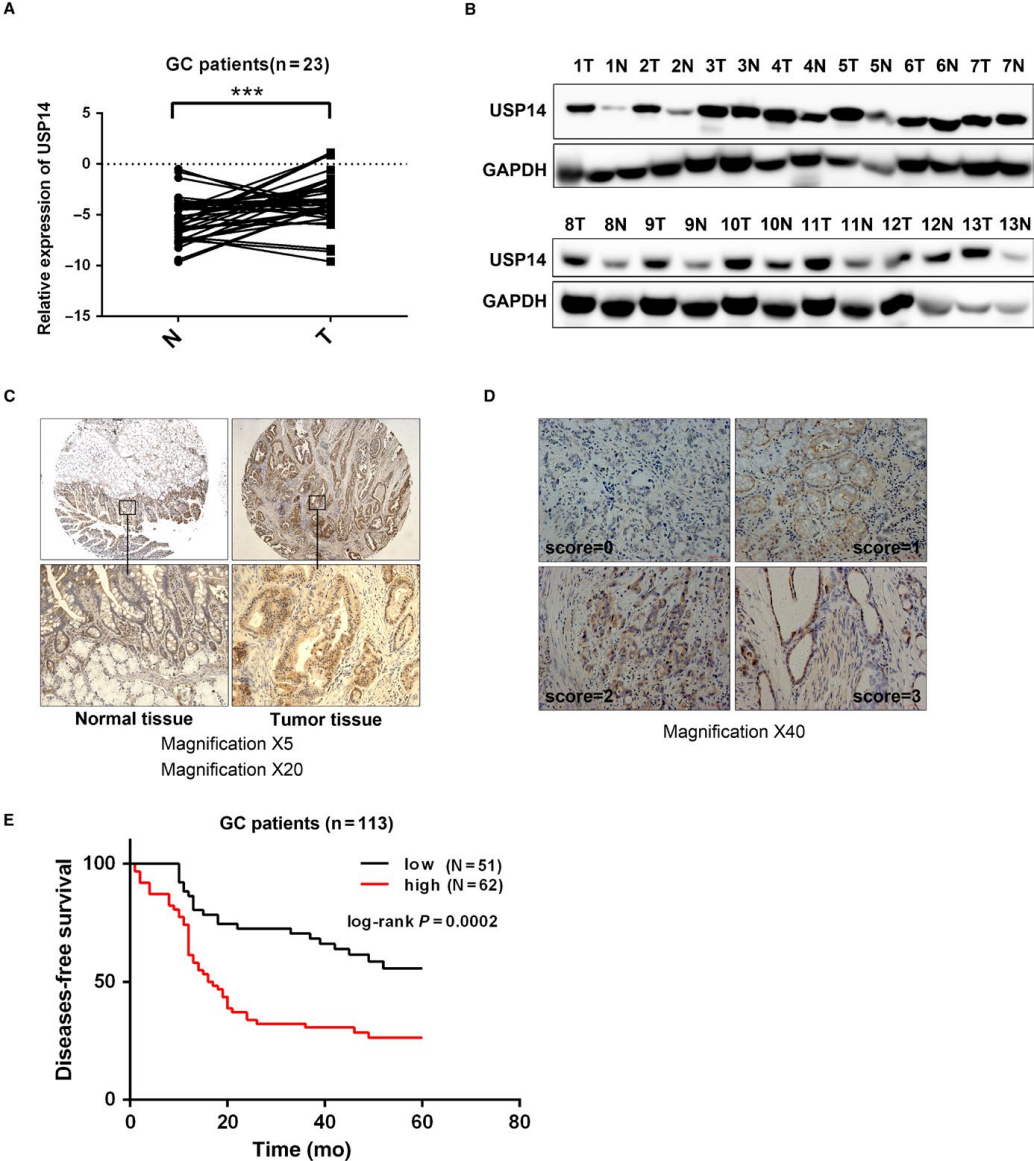
USP14 expression level was increased in GC samples and an independent prognostic marker for DFS in GC. A, Elevated USP14 expression was observed in our own GC samples (N = 23). B, Similarly, USP14 protein level was measured via Western blotting in 13 paired GC and adjacent normal specimens, showing that USP14 protein level were increased in most of the detected cancer tissues (9/13). C, Immunohistochemistry assay showed stronger cytosolic staining intensities of USP14 in GC samples compared with matched normal counterparts (10/15). D, The staining intensities of USP14 in GC samples were scored as 0, 1, 2, and 3, among which 3 indicated the strongest intensity. The representative four staining intensities are shown here. The expression levels of USP14 were determined via multiplying intensities by positive areas. The low expressions of USP14 were 0‐3 points, while the high ones were 4‐12 points. E, GC patients (N = 113) with high USP14 expression levels have an unfavorable DFS (*P *=* *0.0002)

To determine the mRNA level of USP14 in GC, we detected 23 pairs of GC/normal samples, demonstrating that its mRNA levels were increased in the GC samples (Figure 2A). We further tested the USP14 protein level in 13 pairs of GC/normal samples by Western blot, showing that USP14 levels were higher in most GC specimens compared to the adjacent normal tissues (9/13) (Figure 2B). Furthermore, our immunohistochemistry result showed that USP14 was mainly accumulated in the cytosol compartment of malignant gastric tissues (N = 10/15) (Figure 2C). Thus, our results showed that USP14 levels were increased at both mRNA and protein levels in GC samples.

### 3.3 USP14 was an independent DFS marker in GC patients

To evaluate the prognostic value of USP14, we detected the expression of USP14 in 113 archived paraffin‐embedded GC specimens (Table 1). The staining intensities of USP14 were scored as 0, 1, 2, and 3, respectively, for further analysis (Figure 2D). Based on the USP14 level, the total 113 patients was divided into an USP14 low‐expression group composed of 62 patients (54.9%) and an USP14 high‐expression group containing 51 patients (45.1%). Notably, high USP14 expression was associated with the recurrence of the GC patients accepting cisplatin‐based therapy (*P* = 0.0026), suggesting a close relationship between this deubiquitylase and chemotherapies (Table 1). On the other hand, the expression level of USP14 was not correlated with age, gender, differentiation, and TNM stage (UICC).

**Table 1:** Clinicopathologic properties of USP14 expression in 113 pairs of GC specimens

Characteristics	Number of cases	USP14 expression		*P* value
		Low (n = 51)	High (n = 62)	
Age group				0.613
≤60	68	32	36	
>60	45	19	26	
Sex, N				0.3155
Male	93	44	49	
Female	20	7	13	
Differentiation				0.9562
Middle	44	20	24	
Low	69	31	38	
T stage				0.1128
T1/T2	12	8	4	
T3/T4	101	43	58	
Lymph node metastasis				0.1903
Yes	78	32	46	
No	35	19	16	
Distant metastasis				0.6709
Yes	4	2	2	
No	99	39	60	
TNM stage (UICC)				0.1235
I/II	38	21	17	
III/IV	75	30	45	
Infiltrating serous membrane				0.1128
Yes	101	43	58	
No	12	8	4	
Recurrence				0.0026
Yes	64	21	43	
No	49	30	19	

Having confirmed the correlation between USP14 expression and the disease relapse, we then evaluated the value of USP14 in DFS prediction. DFS curves were plotted with the Kaplan‐Meier and compared using the log‐rank method, which gave rise to a result showing that high USP14 expression indicated an unfavorable DFS (*P* = 0.0002) (Figure 2E).

Moreover, the univariate analysis showed that differentiation (*P* = 0.036), tumor size (*P* = 0.016), lymph node metastasis (*P* < 0.001), TNM stage (*P* < 0.001), invasion into serous membrane (*P* = 0.016), and USP14 overexpression (*P* = 0.002) were correlated with DFS, respectively (Table 2). All of these significant factors further underwent the multivariate analysis, which demonstrated that USP14 level was an independent prognostic factor for DFS in GC patients (HR = 3.844, 95% CI = 1.938‐7.624, *P* < 0.001) (Table 3).

**Table 2:** Univariate analysis of DFS for 113GC patients

	*P*	HR	95% CI
Age (≤60, >60)	0.801	1.067	0.645‐1.763
Sex (male, female)	0.992	1.003	0.524‐1.921
Differentiation (low, middle)	0.036	0.566	0.333‐0.964
T (T1‐T2, T3‐T4)	0.016	11.356	1.573‐81.967
N (No, Yes)	<0.001	3.678	1.865‐7.253
M (No, Yes)	0.333	1.776	0.555‐5.682
TNM (I/II, III/IV)	<0.001	4.3	2.179‐8.485
Infiltrating serous membrane (No, Yes)	0.016	11.356	1.573‐81.967
USP14 (low, high)	0.002	2.288	1.351‐3.873

**Table 3:** Multivariate analysis of DFS for 113GC patients

	*P*	HR	95% CI
Differentiation (low, middle)	0.005	2.162	1.268‐3.686
TNM (I/II, III/IV)	0.045	0.577	0.337‐0.987
USP14 (low, high)	<0.001	3.844	1.938‐7.624

### 3.4 USP14 silencing sensitized GC cells to cisplatin in vitro

**Figure 3:**
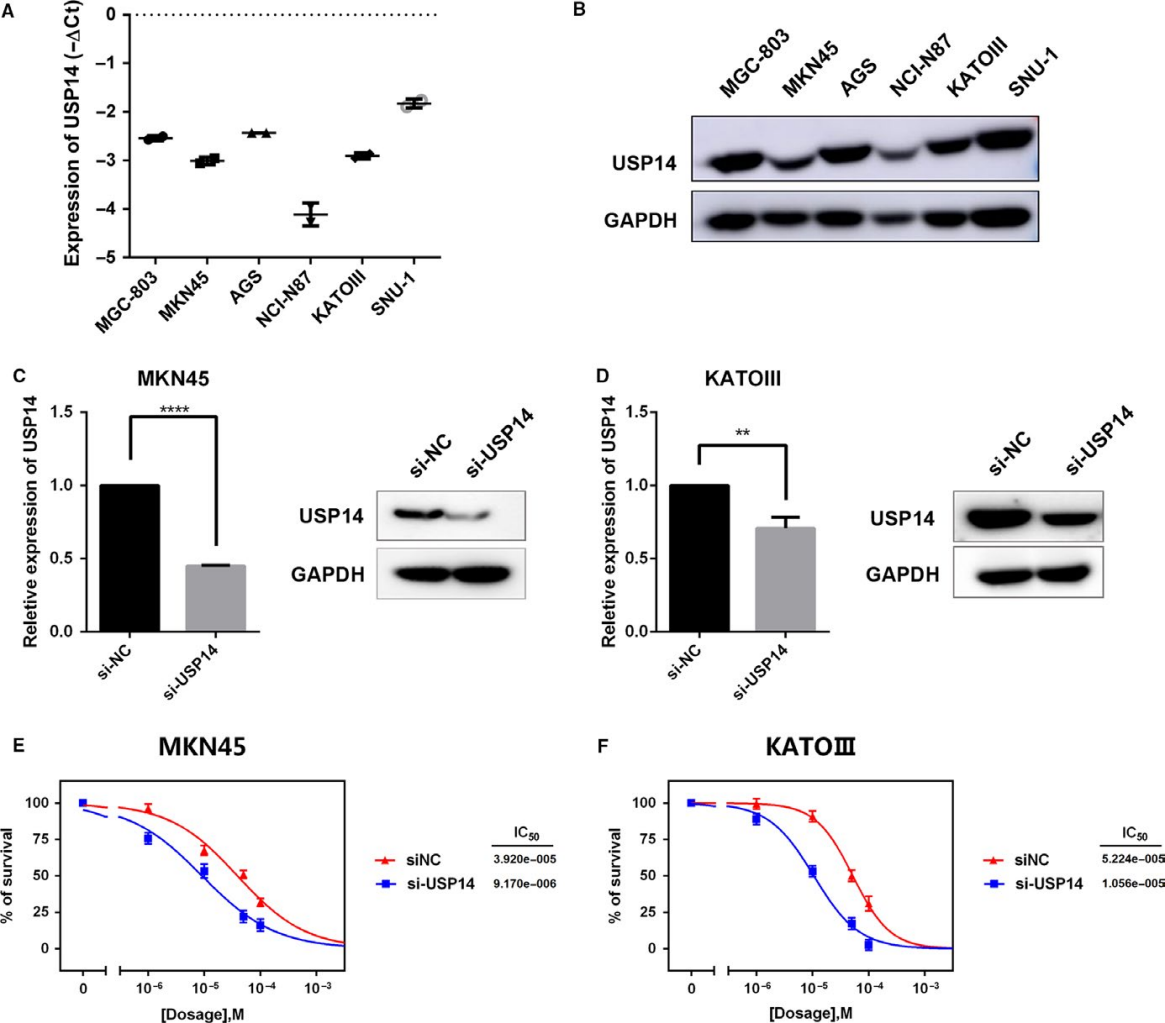
Suppressing USP14 expression sensitized GC cells to cisplatin in vitro. A, B, USP14 has high expression at both mRNA and protein level in the indicated six GC cell lines. C, D, A pool of three siRNAs (100 nmol/L in total) were introduced into MKN45 (C) and KATO III (D) cells, respectively. The USP14 expressions in these cells were measured via RT‐qPCR and western blotting. Data, **P < 0.01, ****P < 0.0001. E, F, USP14 deletion reduced cisplatin IC50 of MKN45 cells from 39.2 ± 5.17 to 9.17 ± 2.68 μmol/L (E). Likewise, cisplatin IC50 decreased from 52.24 ± 8.67 μmol/L to 10.56 ± 5.87 in KATO III cells (F)

As USP14 level was correlated with the survival of GC patients who received cisplatin‐based chemotherapy, we thereby examined whether USP14 played a role in the chemotherapy resistance. In a panel of six GC cell lines (MGC803, MKN45, AGS, NCI‐N87, KATO‐III, and SNU1), high USP14 expressions at both mRNA and protein level were detected in almost all tested cells (Figure 3A,B). These results prompted us to silence USP14 expression to explore its function in GC cells. Pools of three siRNAs (100 nmol/L in total) were used to inhibit USP14 expression in MKN45 or KATO III cells, respectively (Figure 3C,D). As data shown, USP14 silencing significantly decreased the cisplatin IC50 by 4‐5 folds in these cells (Figure 3E,F). Similar results were also observed in AGS and SNU‐1 cells (data not shown). Thus, suppression of USP14 expression greatly enhanced sensitivity of GC cells to cisplatin.

### 3.5 USP14 modulated sensitivity of GC cells to cisplatin through Akt/ERK signaling pathways

**Figure 4:**
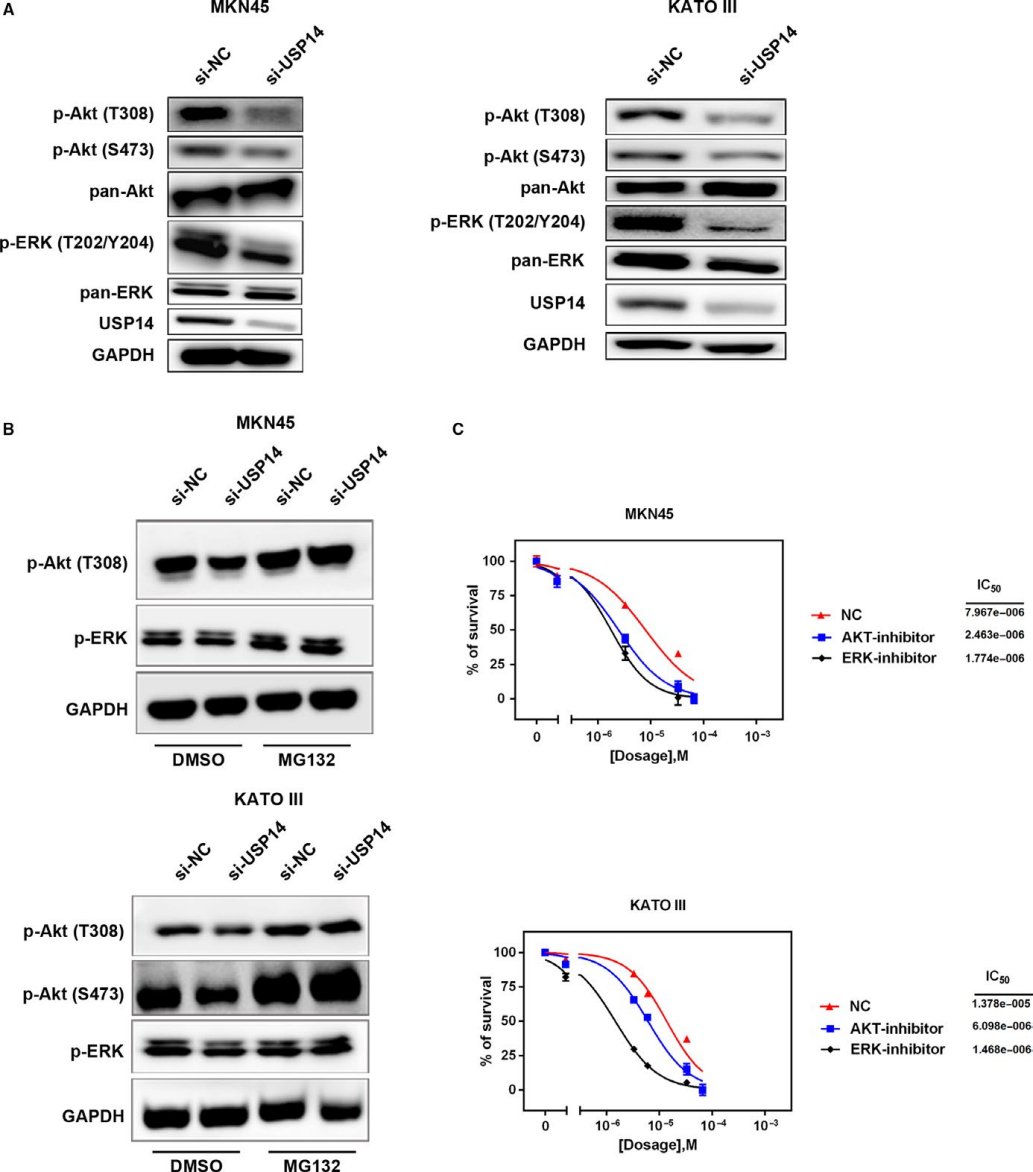
USP14 modulated sensitivity of GC cells to cisplatin through Akt/ERK signaling pathways. A, Blocking USP14 expression significantly reduced p‐Akt (T308/S473) and p‐ERK (T202/Y204) in MKN45 and KATO III cells. One representative result of two independent assays was shown here. B, p‐Akt (T308/S473) and p‐ERK (T202/Y204) were degraded by ubiquitin‐proteasome machinery. Treatment of 10 μmol/L MG132 for 4 h rescued these proteins from degradation in USP14‐deleted MKN45 and KATO III cells. One representative result of two independent assays was shown here. C, Pretreatment of 10 μmol/L LY294002 for 1 h reduced cisplatin IC50 from 7.967 ± 2.89 to 2.463 ± 1.79 μmol/L in MKN45 cells or from 13.78 ± 3.76 to 6.098 ± 2.03 μmol/L in KATO III cells, respectively. Similarly, PD98059 (10 μmol/L, pretreated for 1 h) cisplatin IC50 decreased from 7.967 ± 2.89 to 1.774 ± 0.18 μmol/L in MKN45 cells or from 13.78 ± 3.76 to 1.468 ± 1.85 μmol/L in KATO III cells, these cells which were pretreated with PD98059 for 1 h. DMSO was used as the negative control

USP14 rescues its target proteins from degradation by removing poly‐ubiquitin tags from them. Thus, knockdown of USP14 may lead to impaired rescue of its substrates, which might be responsible for the cisplatin sensitization. We thereby examined the levels of some key factors in survival‐related signaling pathways and found that p‐ERK (T202/Y204) and p‐Akt (T308/S473) were dramatically reduced in USP14‐defitient MKN45 and KATO III cells (Figures 4A and S2). Interestingly, MG132 treatment restored the inhibitory effects on both signaling pathways by USP14 silencing (Figure 4B). These results demonstrated a role of USP14 in preventing p‐ERK (T202/Y204) and p‐Akt (T308/S473) from degradation through proteasome pathway. Moreover, blocking the activation of PI3K/Akt signaling pathway by LY294002 also sensitized GC cells to cisplatin. The IC50 dropped by around 50% upon LY294002 treatment in MKN45 and in KATO III cells. Similarly, the cisplatin sensitization effect was reproduced and even more profound upon the treatment of PD98059, an ERK signaling inhibitor, in those cells (Figure 4C). These results confirmed that inhibition of Akt and ERK signaling pathways could sensitize GC cells to cisplatin. Collectively, inhibition of USP14 facilitated the proteasome‐mediated degradation of activated ERK and Akt, which thereby led to increased sensitivity of GC cells to cisplatin.

### 3.6 USP14 depletion induced apoptosis in cisplatin‐treated GC cells

**Figure 5:**
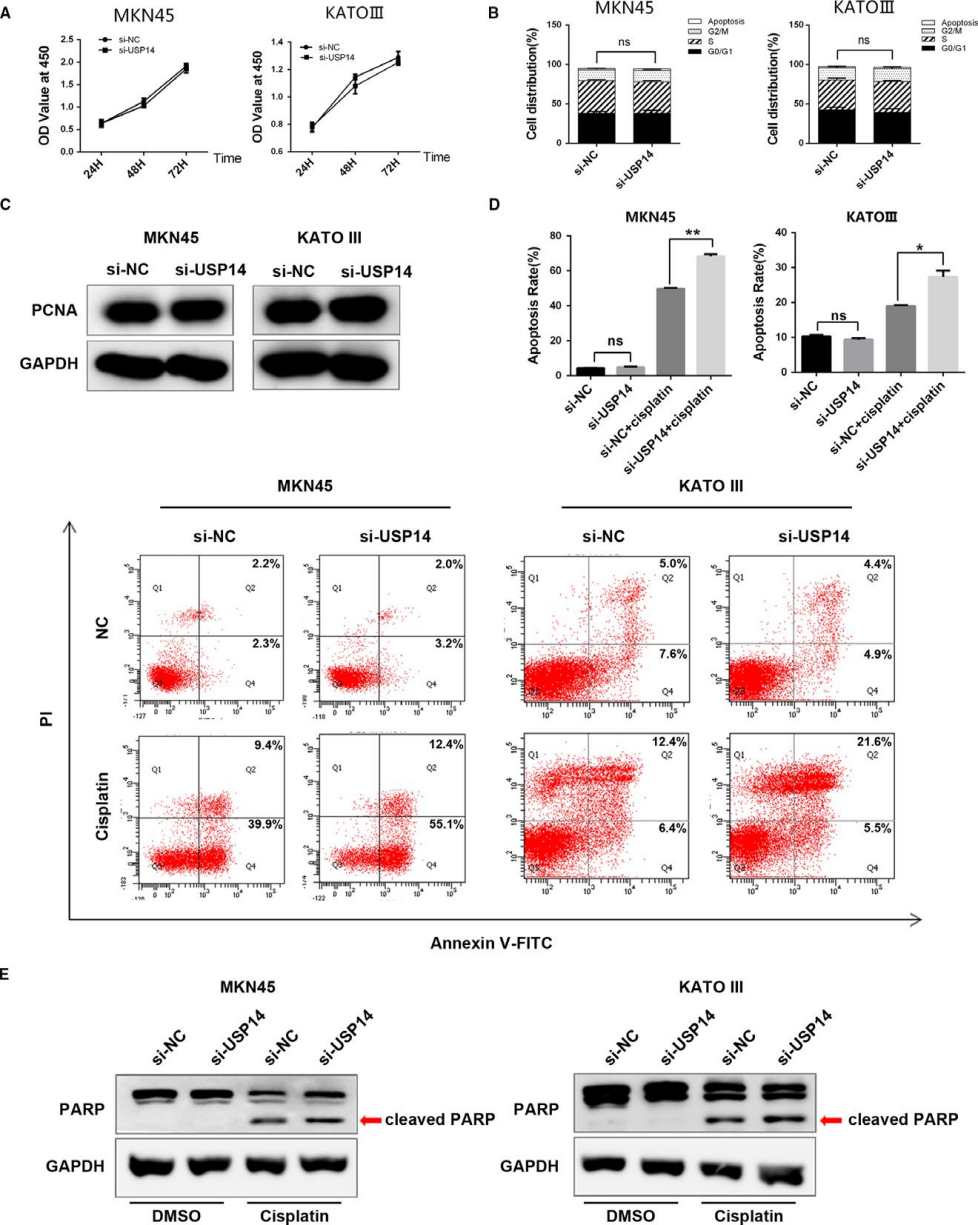
USP14 depletion induced apoptosis in cisplatin‐treated GC cells. A, WST‐1 assay were performed to evaluate the cell viability at 24, 48, and 72 h. Knockdown of USP14 had barely any effect on cell viability in MKN45 and KATO III cells. B, Flow cytometry results showed that the proliferation rate was not affected in USP14 deficient cells. C, Knockdown of USP14 did not affect PCNA expression in MKN45 and KATO III cells. D, Flow cytometry showed that reduced USP14 expression increased the apoptotic population of cisplatin‐treated MKN45 cells by 18.2% or KATO III cells by 8.3%, respectively. E, Depletion of USP14 promoted PARP cleavage in the case of cisplatin dosing. Blocking USP14 expression alone did not increase the cleaved PARP level

As AKT and ERK signaling pathways play important roles in a wide range of cellular activities, there might be multiple biological processes that have contributed to the sensitization of GC cells to cisplatin by USP14 suppression. Thus, we first determined the impact of USP14 silencing on GC cell growth. WST‐1 assay were performed to evaluate the cell viability at 24, 48, and 72 hours. As shown in Figure 5A, knockdown of USP14 had barely any effect on cell viability. We then determined the role of USP14 in cell proliferation. Flow cytometry result showed that the proliferation rate was not affected in USP14 deficient cells, which was further confirmed by the unchanged PCNA levels after silencing of USP14 in both MKN45 and KATO III cells (Figure 5B,C). Next, we explored the role of USP14 in apoptosis. Again, compared to the control cells, we only observed marginal increased apoptotic events in USP14 depleting cells (Figure 5D,E). However, in the presence of cisplatin, knockdown of USP14 elicited a remarkable increase of apoptotic population by flow cytometry, which was validated by the promoted PARP cleavage using western blot (Figure 5D,E). Thus, these results showed that silencing of USP14 alone is not sufficient to cause overt effects on cell growth, proliferation, and apoptosis in GC cells, but could induce significant apoptosis in these cells upon cisplatin treatment. Together, knockdown of USP14 sensitized GC cells to cisplatin through promoting apoptosis in cisplatin‐treated cells via inhibition of Akt/ERK signaling pathways.

## 4 DISCUSSION

Previous studies have shown that USP14 expressions were aberrantly up‐regulated in a variety of malignancies, including multiple myeloma (MM), esophageal squamous cell carcinoma (ESCC), and endometrial cancer (EC).^13^, ^14^, ^15^ The elevated USP14 expressions were associated with poor prognoses of breast cancer.^16^ USP14 could be used as a potential prognostic marker for ESCC and non‐small cell lung cancer patients.^17^, ^18^ In this study, we observed elevated USP14 expressions in GC tissues at both mRNA and protein levels, which was correlated with high recurrence rate in GC patients who accepted cisplatin‐based chemotherapy. Furthermore, we demonstrated that USP14 may serve as an independent DFS marker for GC patients based on the multivariate analysis on a large clinical cohort. This is consistent with previous reports. However, multi‐center‐based investigation is needed for further validation of its predictive value.

It has been shown that silencing of USP14 could block the cell cycle progression and elicit caspase‐dependent apoptosis in MM cells.^13^ Also, inhibition of USP14 led to G0/G1 arrest by accelerating the ubiquitination and degradation of AR in prostate cancer cells.^19^ In addition, small USP14 inhibitors, such as WP1130, b‐AP15, AC17, and pyrithione, could dramatically suppressed cell proliferation and promoted apoptosis in various malignancies.^9^, ^20^, ^21^, ^22^, ^23^, ^24^ However, we did not observe any disturbed biological process, such as cell cycle progression, cell proliferation, and apoptosis, in USP14‐deficient GC cells, although silencing of USP14 dramatically inhibited Akt and ERK signaling pathways. In line with our result, genetic or pharmacological inhibition of USP14 led to reduced phosphorylation of Akt and/or Erk1/2 in hepatocellular carcinoma cells and monocytic leukemia cells.^25^, ^26^ Moreover, inhibition of PI3K/AKT and ERK1/2 signaling pathways could enhance cisplatin sensitivity in urothelial bladder cancer cells and ovarian cancer cells, respectively.^27^, ^28^

As one of three proteasome‐associated DUBs, USP14 plays a key regulatory role in protein proteasomal degradation.^29^ Inhibition of USP14 activity would lead to a dysfunctional proteasome, eliciting a broad accumulation of ubiquitinated proteins. However, in this study, we found that the levels of p‐Akt (T308/S473) and p‐ERK (T202/Y204) were decreased in response to USP14 suppression, suggesting a proteasome‐free deubiquitination activity of USP14 involving in the regulation of phosphorylated AKT and ERK levels. MUL1 and TTC3 have been reported to be the E3 ubiquitin ligases responsible for the proteasomal degradation of p‐Akt via K48‐linked ubiquitination.^30^, ^31^ Meanwhile, MEKK1 has recently been found to function as an E3 ligase through its PHD domain to promote ubiquitin/proteasome‐mediated degradation of phosphorylated ERK1/2, besides acting as an upstream activator of the ERK pathway through its kinase domain.^32^ Thus, we speculate that USP14 may counteract the ubiquitination processes mediated by these E3 ubiquitin ligases through its proteasome‐free deubiquitination activity, whereas inhibition of USP14 discharges this “ubiquitin‐chopping” protective mechanism, resulting in the degradation of p‐Akt and p‐ERK.

Here, we have demonstrated that inhibition of USP14 sensitized GC cells to cisplatin through impeding Akt and ERK signaling pathways that involve in a diversity of biological processes. Our experimental assays further revealed that promoting apoptosis in cisplatin‐treated cells plays a critical role in sensitization of these cells to cisplatin. Besides, other mechanisms such as autophagy may also involve in this sensitization process. It has been well documented that autophagy plays a dual role in chemotherapeutic resistance. On the one hand, autophagy clears damaged organelles and thus protects cancer cells from chemodrugs. On the other hand, excessive autophagy in apoptosis‐deficient cells could trigger massive cell death, thereby sensitizing cancer cells to the chemodrugs.^33^ Previous study has shown that USP14 could negatively regulate autophagy by removing ubiquitin chains of Lys63 from Beclin‐1.^34^ Thus, to explore the role of USP14 in autophagy in GC cells, we determined the levels of LC3A/B I and LC3A/B II in USP14‐depleted MKN45 and KATO III cells, and observed a high ratio of LC3A/B II to LC3A/B I expression, indicating these cells were undergoing autophagy (Figure S3). Further investigation is warranted to reveal the multifaceted functions of USP14 and the mechanisms underlying chemotherapeutic sensitization of GC cells.

## 5 CONCLUSION

In this study, we observed elevated USP14 expressions in GC tissues, which may serve as an independent poor prognostic marker for DFS in GC patients. Inhibition of USP14 sensitized GC cells to cisplatin by triggering apoptosis in cisplatin‐treated cells via impeding Akt and ERK signaling pathways. These results shed lights on a novel strategy of combining USP14 inhibition with the cisplatin‐based chemotherapy to improve the outcome of GC patients.

## CONFLICT OF INTEREST

None declared.
