# Supplementary figures and images for: USP14 as a novel prognostic marker promotes cisplatin resistance via Akt/ERK signaling pathways in gastric cancer
Source: Cancer Med. 2018 Sep 17;7(11):5577–88. doi: 10.1002/cam4.1770 (PMC6246950; doi:10.1002/cam4.1770)

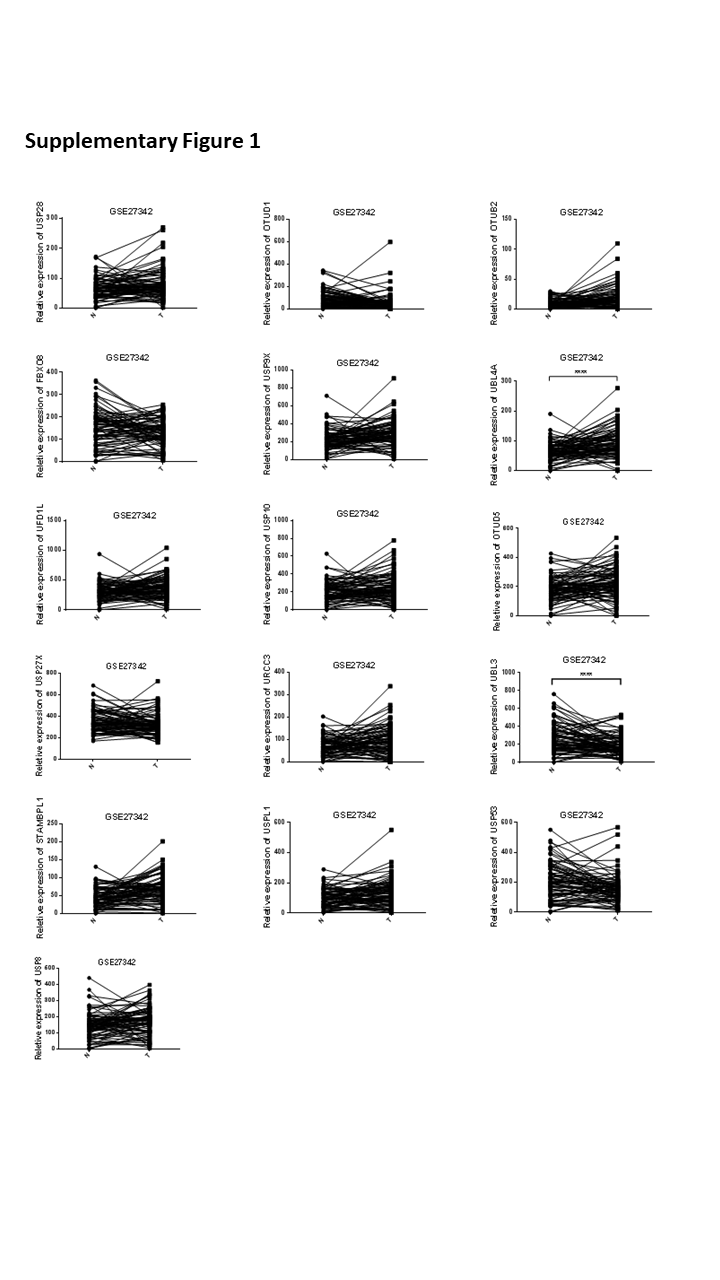

Supplement: Supplementary file 1 [file CAM4-7-5577-s001.TIF]

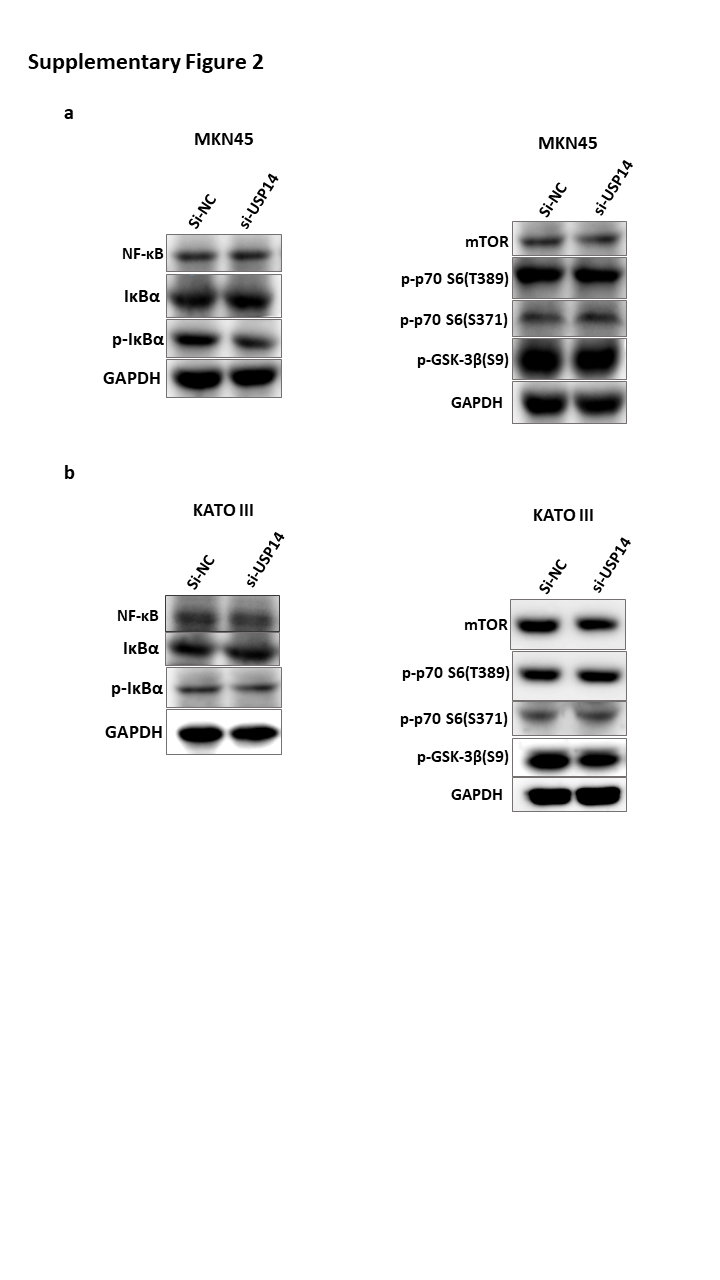

Supplement: Supplementary file 2 [file CAM4-7-5577-s002.TIF]

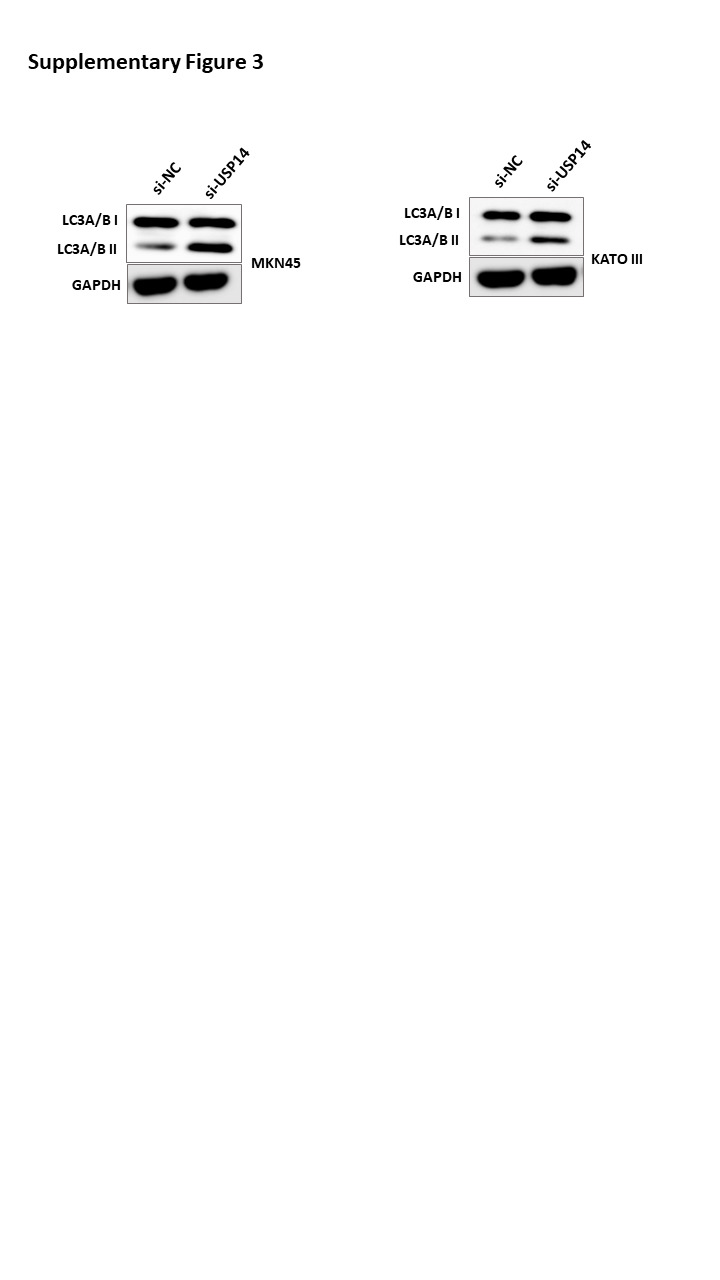

Supplement: Supplementary file 3 [file CAM4-7-5577-s003.TIF]
